# Prospects for regenerative medicine approaches in women's health

**DOI:** 10.1111/joa.12336

**Published:** 2015-07-15

**Authors:** Katja Schenke‐Layland, Sara Y. Brucker

**Affiliations:** ^1^ Department of Women's HealthResearch Institute for Women's HealthUniversity Hospital of the Eberhard Karls UniversityTübingenGermany; ^2^ Department of Cell and Tissue EngineeringFraunhofer Institute for Interfacial Engineering and Biotechnology (IGB)StuttgartGermany; ^3^ Department of Medicine/CardiologyCardiovascular Research LaboratoriesDavid Geffen School of Medicine at UCLALos AngelesCAUSA; ^4^ Department of Women's HealthUniversity Women's Hospital of the Eberhard Karls UniversityTübingenGermany

**Keywords:** cervix, genital reconstruction, Mayer–Rokitansky–Küster–Hauser syndrome, stem cells, tissue engineering

## Abstract

Novel regenerative strategies, stem cell‐based therapies or the development of advanced human cell‐based *in vitro*‐manufactured preclinical test systems offer great potential to generate advances in clinical practice in the field of women's health. This review aims to provide a brief overview of the current advances in the field.

## Introduction

The development of new regenerative therapies, adapted where relevant to the differing needs of men and women, with the potential to address inherited or acquired, acute or chronic, as well as common or rare diseases, has been challenging. In particular, translating basic knowledge in regenerative medicine into the clinic represents a major obstacle.

**Figure 1:**
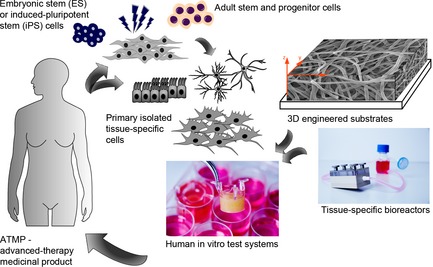
Schematic depiction of tissue engineer‐ing concept.

The concept of tissue engineering has the potential to revolutionize the field of regenerative medicine, offering an answer to the problems of increasing donor organ shortage or providing improved humanized *ex vivo*‐manufactured *in vitro* test systems (Schenke‐Layland & Nerem, 2011). Tissue engineering integrates discoveries from multiple fields, including cell, matrix and developmental biology, biochemistry and chemistry, materials sciences, physics, medicine and biotechnology with the aim to manufacture complex three‐dimensional (3D) tissue and organ structures that can serve either as vital implants [advanced‐therapy medicinal products (ATMPs)] or as off‐the‐shelf or patient‐tailored *in vitro* organoid test systems (Pusch et al. 2011; Fig. 1). The production of an engineered tissue requires the use of appropriate cells and substrates. The main success in this field has come from the use of primary cells, isolated from patients, that were seeded onto 3D scaffolds or matrices in order to produce extracellular matrix (ECM) resembling that of the native tissue (Howard et al. 2008); however, this strategy has its limitations. Although the combination of tissue engineering concepts with stem cell‐based approaches holds much promise for major advancements in the field of regenerative medicine (Howard et al. 2008), caution is advised when interpreting research data for the public. There has been much debate over the hope and hype of stem cell research and tissue engineering over the last decade (Nerem, 2006; Lo & Parham, 2009; Oerlemans et al. 2014). The promise that stem cell research will soon lead to general cures for diseases such as Parkinson's disease, spinal cord injury, heart disease or diabetes has so far not been kept. Nevertheless, a variety of successful stem cell‐based applications have either found their way into clinical reality, or they have led to breakthrough research findings that will help improve therapeutic strategies (Ilic & Polak, 2011).

## Stem cell‐based therapeutic strategies in women's health

The hope that many diseases may someday be treated using stem cells is inspired by the historical success using adult stem cells derived from bone marrow to treat patients with leukemia and other cancers, or inherited blood disorders (Lo & Parham, 2009; Ilic & Polak, 2011). However, there are a variety of stem cells with a diverse differentiation potential based on which they can be classified. The three groups of stem cells that are most relevant for a potential application in regenerative medicine are pluri‐, multi‐ and unipotent stem cells.

Pluripotent stem cells have the potential to self‐renew and differentiate into any of the three germ layers: ectoderm, endoderm and mesoderm that give rise to all tissues and organs of the human body. Embryonic stem (ES) cells are currently the only known natural occurring pluripotent stem cells (Ilic & Polak, 2011). As indicated by their name, induced‐pluripotent stem (iPS) cells also belong to this group (Ilic & Polak, 2011). iPS cells are derived from somatic, tissue‐specific cells that can be reprogrammed using defined factors to form pluripotent stem cells (Schenke‐Layland et al. 2008). Early human iPS cell lines were derived by inserting genes encoding for transcription factors using retroviral vectors (Takahashi et al. 2007; Yu et al. 2007; Park et al. 2008). Since then, researchers have successfully addressed safety concerns about inserting oncogenes and insertional mutagenesis, realizing reprogramming without known oncogenes using adenovirus vectors rather than retrovirus vectors or using a plasmid with a peptide‐linked reprogramming cassette (Lo & Parham, 2009). The successful reprogramming of human somatic cells into a pluripotent ES cell‐like state provides a method to generate customized, potentially patient‐specific pluripotent cells for regenerative medicine efforts. However, this assumes that iPS cells possess a differentiation potential similar to that of ES cells, and critical study of the differentiation behavior of iPS cells will be essential for iPS cell‐based therapies to become clinical reality (Schenke‐Layland et al. 2008).

Multipotent stem cells can also self‐renew; however, they are restricted in their differentiation potential to certain cell lineages (Votteler et al. 2010). Unipotent stem cells are the least potent. They can self‐renew and differentiate into only one cell type (Ilic & Polak, 2011). Mesenchymal stem cells (MSCs) are a typical example of multipotent stem cells. MSCs are unspecialized mesodermal cells that reside within niches in various tissues of the human body, including bone marrow, skin, intestine, adipose tissue or the developing human heart (Votteler et al. 2010; Schenke‐Layland et al. 2011). The main biological function of multi‐ and unipotent stem cells is to replenish dying cells, therefore contributing to the regeneration of diseased or damaged tissues, as well as to ensure normal cell turnover of renewable tissues (Votteler et al. 2010).

In the field of women's health, various pluri‐ and multipotent stem cells have found their application in research approaches. Recent comprehensive reviews have critically analyzed the potential to use stem cells to treat infertility (Hayashi et al. 2012; Bhartiya et al. 2014; Volarevic et al. 2014). Specifically, the potential to achieve ovarian regeneration and oocyte production utilizing either a rare population of cells that reside in human ovaries (White et al. 2012) or pluripotent stem cells (Hayashi et al. 2012; Bhartiya et al. 2014) is both promising and exciting. However, inefficient cell derivation and differentiation protocols, severe epigenetic and genetic changes associated with extensive *in vitro* manipulation, and ethical and regulatory constraints represent major challenges (Bhartiya et al. 2014), heating‐up the current debate over a clinical translatability of these studies. Nevertheless, the idea to develop novel *in vitro* ‘disease‐in‐a‐dish’ models (Hayashi et al. 2012) or female reproductive tract mimics (Laronda et al. 2013) utilizing human ES and iPS cells in order to understand the precise molecular pathologies of infertility, and to design new treatment strategies is highly exciting. For example, King and colleagues have successfully used alginate hydrogels to create a mouse 3D ovary and oviduct culture system that can be potentially used to study mechanisms of ovarian cancer development (King et al. 2011). It will be interesting to see how this system can be transferred into the human system.

Stem cells have also been thought to either protect or contribute to various diseases in women. For example, it has been suggested that declining levels of endogenous estrogen in conjunction with age can contribute to a significant endothelial dysfunction and a downregulation of the numbers of circulating endothelial progenitor cells (EPCs) in postmenopausal women, which may contribute to coronary artery disease (CAD; Hutter et al. 2012). Therefore, it has been speculated that EPCs play a main role for cardiovascular health, and EPC number assessment could be used as a biomarker for CAD diagnosis (Hutter et al. 2012). Other studies have identified stem and progenitor cells that can be responsible for normal tissue regeneration but also for pathological proliferative disorders of the human endometrium, which is a highly dynamic tissue of mesodermal origin that constitutes the mucosal lining of the fused Mullerian ducts of the uterus that undergo cycles of growth and regression with each menstrual cycle (Oliveira et al. 2012 Ferenczy & Bergeron, 1991; Gargett et al. 2009; Ye et al. 2011; Yang & Huang, 2014). Endometriosis is an endometrium‐associated disease that is manifested by the presence of both endometrial glandular and stromal cells outside the uterus (Králíčková et al. 2014). While there have been many hypotheses about the disease onset and progression, no theory by itself explains all types of described endometriotic lesions (Maruyama, 2014). It is therefore likely that multiple mechanisms lead to endometriosis, including translocated endometrial stem and progenitor cells (ESPCs). It will be highly interesting to see if patient cells can be utilized to establish 3D *in vitro* ‘disease‐in‐a‐dish’ test systems with the aim of shedding further light onto the mechanisms of how endometriosis develops and can change into several types of cancer.

## Tissue engineering strategies in women's health

As described in the previous section, many groups have shown that the endometrial tissue contains stem and progenitor cells that have a regenerative capacity. Verdi et al. (2014) have comprehensively reviewed how these cells were utilized to date for applications in tissue engineering and regenerative medicine. For example, when seeded on scaffolds using artificial meshes, ESPCs were used for the treatment of pelvic organ prolapse (Edwards et al. 2013), and were tested *in vivo* on a rat skin wound repair model in which ESPCs lead to a promoted neovascularization, increased tissue integration, reduced chronic inflammation and an increased deposition of collagen fibers (Ulrich et al. 2012). Shoae‐Hassani et al. (2013) have shown that ESPCs could be differentiated into smooth muscle cells on bioabsorbable polyethylene‐glycol‐ and collagen‐containing hydrogels. The authors claim to further utilize their culture system in order to engineer a urinary bladder wall for the application in women, which is an important clinical area considering that about 400 million people worldwide are afflicted with urinary bladder disease (Shoae‐Hassani et al. 2013). Oerlemans et al. (2013) have recently re‐analyzed the role of tissue‐engineered products for the treatment of urological defects. They concluded, that many promising tissue engineering efforts are currently conducted with focus on the urinary system – some of which have started to enter clinical practice, others need more research efforts before they can be applied safely for human disorders, especially with focus on women and children.

Another important area for the potential application of tissue‐engineered constructs in women's health is genital reconstructive surgery. Congenital and acquired malformations can adversely affect the normal anatomy of the female reproductive tract. Genital malformations have an incidence of 0.1–5% in the general female population (Oppelt et al. 2005). In addition to uterine malformations, vaginal and cervical abnormalities, and malformations of the adnexa have been described, although there are several classification systems available, with the latest from the ESHRE and ESGE trying to describe the complexity of female genital malformations (Grimbizis et al. 2013). Rare congenital genital malformations that are acquired during embryonic development, such as Mayer–Rokitansky–Küster–Hauser (MRKH) syndrome, occur in approximately 1 in 4500 female live births (Rall et al. 2013). While there exist a few reports of family cases, the majority of MRKH cases occur randomly throughout the general population (Morcel et al. 2007). To date, the etiology of MRKH syndrome still remains unclear. There are varying phenotypes of MRKH syndrome. Sometimes, associated malformations such as upper urinary tract (~40%) or skeletal abnormalities (~30–40%) can be diagnosed (Morcel et al. 2007). In order to restore the native anatomy of the vagina, allowing a sexual function and therefore potentially improving the patient's quality of life, various reconstructive surgery procedures have been proposed (Brucker et al. 2011; Raya‐Rivera et al. 2014). Moreover, a tissue‐engineered vaginal replacement strategy has been demonstrated in an autologous rabbit model (Dorin et al. 2011). In this study, Dorin and colleagues used primary isolated vaginal epithelial and smooth muscle cells that were seeded onto tubular scaffolds made of polyglycolic acid (Dorin et al. 2011). They then implanted the scaffolds into the vaginal position in the cell donor rabbits, and after 6 months post‐implantation, the authors found neovaginas that closely resembled the native vaginal tissue in regards to its histoarchitecture and function (Dorin et al. 2011). Nevertheless, based on the currently available data and the current authors' own clinical experience, the advantage of a tissue‐engineered vagina over the laparoscopic‐assisted creation of a neovagina cannot be seen (Rall et al. 2014), as major problems remain with the tissue‐engineered constructs, including tissue shrinkage.

**Figure 2:**
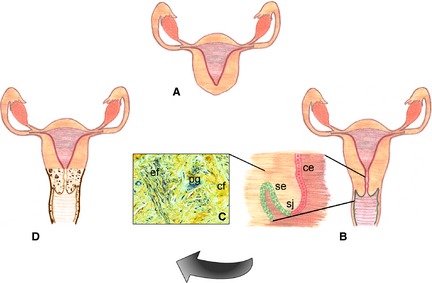
(a) Schematic depiction of a functional corpus uteri without a cervix. (b) Investigation of natural cervix structures, including columnar epithelial (ce), squamocolumnar junctional (sj) and squamous epithelial (se) cells, as well as (c) ECM components such as proteoglycans (pg), collagen and elastic fibers (cf and ef) will provide crucial information for (d) the blueprint design of *ex vivo*‐engineered cervical implants.

Other investigators, including the authors' own groups, have focused on the engineering of cervix or uterus structures, either to design advanced human‐based *in vitro* test systems or to create implantable ATMPs for patients who, for example, have only a vaginal dimple and functional uterine body (corpus uteri) without a cervix (Fig. 2a), although it is a great challenge to keep fertility with an anastomosis between the reconstructed neovagina and the corpus uterus. House et al. (2010) reported on the successful creation of a cervical‐like construct made of silk scaffolds and human primary isolated human cervical cells. Lü et al. (2009) reported on the *in vitro* reconstruction of a uterine tissue containing a smooth muscle layer that could be implemented in an advanced research *in vitro* system. The current authors' own strategy is to identify the detailed blueprint of the natural normal human cervix structures, defining crucial cellular matrix and ECM elements, as well as biomechanical properties that will allow to eventually design a clinically relevant and fully functional cervix replacement (Fig. 2).

## Concluding remarks

Although much progress has been made in the fields of stem cell research for the development of therapies and tissue engineering for application in regenerative medicine in women's health, the translation into clinical practice has been limited as no tissue‐engineered vagina, cervix or uterus has found its way into routine therapies. Nevertheless, stem cell‐ or ECM‐based tissue engineering strategies have a great potential for regenerative medicine. That potential offers hope to millions of future patients who have diseases for which existing treatments are inadequate or, in many cases, are non‐existing.

## Conflict of interest

No conflict of interest needs to be declared.

## Author contribution

K.S.L. and S.B. drafted, critically revised and approved the article.
